# Glacial History of the North Atlantic Marine Snail, *Littorina saxatilis*, Inferred from Distribution of Mitochondrial DNA Lineages

**DOI:** 10.1371/journal.pone.0017511

**Published:** 2011-03-11

**Authors:** Marina Panova, April M. H. Blakeslee, A. Whitman Miller, Tuuli Mäkinen, Gregory M. Ruiz, Kerstin Johannesson, Carl André

**Affiliations:** 1 Department of Marine Ecology – Tjärnö, University of Gothenburg, Strömstad, Sweden; 2 Marine Invasions Laboratory, Smithsonian Environmental Research Center, Edgewater, Maryland, United States of America; 3 South African Institute for Aquatic Biodiversity, Grahamstown, South Africa; Biodiversity Insitute of Ontario - University of Guelph, Canada

## Abstract

The North Atlantic intertidal gastropod, *Littorina saxatilis* (Olivi, 1792), exhibits extreme morphological variation between and within geographic regions and has become a model for studies of local adaptation; yet a comprehensive analysis of the species' phylogeography is lacking. Here, we examine phylogeographic patterns of the species' populations in the North Atlantic and one remote Mediterranean population using sequence variation in a fragment of the mitochondrial cytochrome *b* gene (607 bp). We found that, as opposed to many other rocky intertidal species, *L. saxatilis* has likely had a long and continuous history in the Northwest Atlantic, including survival during the last glacial maximum (LGM), possibly in two refugia. In the Northeast Atlantic, several areas likely harboured refugial populations that recolonized different parts of this region after glacial retreat, resulting in strong population structure. However, the outlying monomorphic Venetian population is likely a recent anthropogenic introduction from northern Europe and not a remnant of an earlier wider distribution in the Mediterranean Sea. Overall, our detailed phylogeography of *L. saxatilis* adds an important piece to the understanding of Pleistocene history in North Atlantic marine biota as well as being the first study to describe the species' evolutionary history in its natural range. The latter contribution is noteworthy because the snail has recently become an important model species for understanding evolutionary processes of speciation; thus our work provides integral information for such endeavours.

## Introduction

Recent biogeographic histories of arctic and north-temperate species demonstrate a strong influence of climate variation during glacial periods of the Pleistocene Epoch [1]–[5]. Distributions of north-temperate terrestrial species have undergone repeated cycles of contraction and expansion during glacial periods, and present-day distributions are believed to be the result of expansion from southern refugia after the Last Glacial Maximum (LGM) ∼18–25 kya [2], [3]. Distributions of rocky shore species in the North Atlantic also contracted and became fragmented during the LGM since large coastal areas were covered by ice; even still, suitable rocky shore habitats, ice-free or ice-covered only seasonally, probably existed even at higher latitudes (for ex, Iceland - Faeroes region and Southwest Ireland; see maps in [6] and [7]).

In the Northwest Atlantic (NWA), the southern margin of the ice sheet was near Long Island during the LGM [6], [8], which also coincides with the southern border of rocky shore habitats. As such, it has been assumed that obligate rocky shore species likely went extinct during the LGM since habitat south of Long Island would not have been conducive to survival [8]. Thus many rocky intertidal species may have naturally re-colonized the Northwest Atlantic from the Northeast Atlantic (NEA) following glacial retreat [9], [10] - indeed, in a comparative phylogeographic study, Wares & Cunningham concluded that five of six rocky intertidal species showed signs of expansion from Europe to North America after the glaciers receded [8]. However, recent ice-sheet reconstructions suggest that Maritime Canada (e.g., Newfoundland and Nova Scotia) may actually have been ice-free rocky shore habitats during the LGM [11], [12]; thus some species could have potentially survived the LGM in these refugia. In NEA, species are also believed to have survived the LGM in glacial refugia, and suitable refugial locations may have included the Azores, the Mediterranean and Iberian coasts, the English Channel (the Hurd Deep and the coast of Brittany), Southwest Ireland at the edge of the Eurasian ice sheet [6], [7], and potentially Iceland and the Faeroes [8], [13], [14]; but see [15].

The rough periwinkle snail *Littorina saxatilis* is highly abundant and widely distributed across the North Atlantic, thus presenting a good model species to explore phylogeographic patterns in the region. *Littorina saxatilis* exists in a myriad of habitats, including exposed cliffs, rocky shore boulders and macroalgae, as well as salt marshes and mudflats, and is naturally distributed in populations throughout the North Atlantic: from the Barents Sea to Portugal in NEA, North Atlantic islands (the Faeroes, Iceland and Greenland), and from Baffin Island to Delaware Bay in NWA [16] (though recent surveys have not found the snail south of Long Island; A. Blakeslee, pers. obs.). There are also two cryptogenic populations in the Mediterranean: in lagoons around Venice and in the Gulf of Gabés in Tunisia [16]. These Mediterranean populations may be relicts of a wider Pleistocene distribution, when, based on fossil records, *L. saxatilis* lived possibly as far south as Gibraltar and the coast of Morocco; alternatively, they are the result of more recent introductions [16].

In addition to a wide latitudinal range and a broad ecological spectrum, *L. saxatilis* exhibits extreme variation in shell morphology, and distinct ecotypes can be found in many locations [16]. Such striking variations in morphology are believed the result of local adaptations facilitated by limited dispersal of the species: *L. saxatilis* possesses an ovoviviparous reproductive strategy (lacking oceanic larval dispersal), and adult snails have limited home-range dispersal (∼2–10 meters in their lifetimes; [17]). For example, in three regions, Galicia (Spain), the United Kingdom, and in Sweden, morphologically divergent ecotypes, adapted to crab predation or wave exposure live in sympatry, thus providing an excellent model for studies of evolutionary divergence and ecological speciation [18], [19]. However, even though *L. saxatilis* is a well-studied species, phylogenetic relationships among these populations, or other geographic populations of the species, have remained completely unknown.

In the present study, we reconstruct the phylogeography of *L. saxatilis* in the North Atlantic using mitochondrial DNA variation to distinguish between alternative hypotheses among the following scenarios: (1) *L. saxatilis* survived the LGM in refugia in the NWA vs. extinction and later re-colonization from Europe; (2) phylogenetic connections in present-day NEA populations originate from one common refugium vs. colonisations from several refugia; and (3) the Venetian population (one of the two cryptogenic populations in the Mediterranean) is a relict population vs. a recent anthropogenic introduction. To determine the relationship of our model organism among other North Atlantic rocky intertidal species with broad distributions, we also compared our results for *L. saxatilis* with existing phylogeographic evidence of other North Atlantic species.

## Materials and Methods

### Sampling, DNA extraction, and cytochrome *b* sequencing

**Figure 1:**
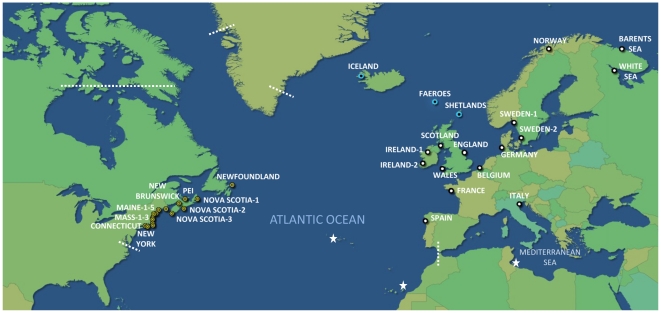
Map of sampled populations of *L. saxatilis* across the North Atlantic. Dataset includes 16 sites in the Northwest Atlantic (“NWA”; yellow circles), 3 sites from North Atlantic islands (“ISL”, blue circles), and 15 sites from Northeast Atlantic (“NEA”; black circles). Dashed lines show the northern and southern ranges of the species' distributions in NWA and the southern range of the distribution in NEA (the northern range in NEA is Svalbard and Novaya Zemlya which is beyond the scope of our map); white stars denote additional isolated populations of the species. Data for the species' North Atlantic range is derived from [16].

We obtained 34 samples covering most of the species' natural range, including a cryptogenic Mediterranean sample from Venice, Italy (Fig. 1, Table S1; hereafter referred to as “populations”). For many of our analyses, the pool of sampled North Atlantic populations was divided into three regions: “NWA” - Northwest Atlantic, “NEA” - Northeast Atlantic mainland, and “ISL” - North Atlantic Islands, as shown in Fig. 1. In total, we analyzed 778 individuals, 7–47 per population (Table S1).

In three of our populations (Spain, England, and Sweden-1; Fig. 1), the species exists as two distinct ecotypes (reviewed by [19]). For these populations, our samples comprise both ecotypes in equal numbers. Statistical comparisons between the ecotypes did not reveal significant differentiation (variation among ecotypes within sites using a hierarchical analysis of molecular variance: *Φ*sc = 0.07, *p* = 0.07); therefore, we pooled ecotype data for all further analyses. Detailed analysis of genetic variation between sympatric ecotypes of *L. saxatilis* is the focus of another on-going study and will not be discussed further here. Furthermore, in four areas (UK, Brittany, Norway, and the Barents Sea), *L. saxatilis* lives in sympatry with two closely related and morphologically similar species, *L. compressa* and *L. arcana*. In these areas, we only sampled mature, brooding females, ensuring accurate identification since *L. saxatilis* is the only ovoviviparous species in its genus.

DNA was extracted from the snail's foot using a BioSprint 96 DNA Blood kit (Qiagen Inc., Germantown, MD, USA) or a standard CTAB method. We amplified a 669 bp fragment of the mitochondrial cytochrome *b* gene, using the forward primer TTCCCGCACCTTCAAATCTT [20] and reverse primer GGACTAGGGCCGAAAGTATAAATA [21]. PCR was performed in 50 µl reaction volumes containing 20 ng template DNA, 1 X reaction buffer, 2 mM MgCl2, 0.25 mM dNTPs, 0.5 µM forward and reverse primers and 1.25 U *Taq* polymerase. PCR cycling conditions were 95° for 2 min; 29 cycles of 95° for 45 s, 55° for 45 s, and 72° for 45 s, and a final 2 min-extension at 72°. Sequencing was performed at the Smithsonian Institution's Laboratory of Analytical Biology (Suitland, Maryland, USA) and at Macrogen Inc. (Seoul, South Korea).

### Alignment and phylogenetic analyses

Sequencing was performed in both forward and reverse directions and sequences were assembled and manually inspected for ambiguities in Geneious 4.8.4 and Sequencher 4.8. All sequences were trimmed to a final length of 607 bp. Sequences were aligned without gaps using the ClustalW algorithm [22] and collapsed into haplotypes using TCS v.1.21 [23]. We also constructed rarefaction curves using EstimateS 8.0 [24] to estimate haplotype diversity in each population and to quantify the effects of sampling effort on resulting haplotype diversity (as in [25]).

The optimal nucleotide substitution model was selected based on the Akaike Information Criterion in Modeltest 3.7 [26]. The selected model was then used in Bayesian phylogeny reconstructions using the software MrBayes 3.1.2. [27]. Trees were rooted with a cytochrome *b* sequence from *L. fabalis* (accession number U46808, [28]). We partitioned the data by codon position and performed two independent runs with five chains per run, sampling trees every 100 generations for 2 million generations. Convergence and appropriate burn-in interval were assessed by examining the plot of likelihood scores and comparing average standard deviation of split frequencies between runs. One thousand trees were discarded as burn-in and the remaining 19 000 trees were used to produce a 50%-majority rule consensus tree with posterior probability for each node.

### Estimation of divergence times between haplotypes

For haplotype clades, identified in the phylogenetic analyses, times to most recent common ancestor (TMRCA) were obtained by the Bayesian Markov chain Monte Carlo method, implemented in BEAST v.1.5.2 [29] based on two calibration points from fossils and allozyme divergence data. We chose this calibration method instead of using a molecular clock for cytochrome *b* because the latter is itself an estimate that was originally based on fossil evidence. Calibration points were: 1) divergence time between *L. fabalis* and the “*saxatilis*” species complex, including *L. saxatilis*, *L. compressa* and *L. arcana*, that occurred between 4–3.5 Ma (the colonization of the North Atlantic by the common Pacific ancestor after the opening of the Bering strait) and 2 Ma (fossil records of *L. fabalis* and *L. islandica* - the extinct ancestor of *L. saxatilis* and *L. arcana*; [16]); and 2) 0.06–1 Ma estimates of divergence time between *L. saxatilis* and *L. arcana* obtained from allozyme variation ([28], calculated on Nei's D genetic distance from [30]). Our dataset for the analyses in BEAST included the cytochrome *b* sequence from *L. fabalis* (accession number U46808, [28]) and haplotype 26 (accession number JF340319) typically found in *L. arcana*. This haplotype belongs to a clade that is characteristic of *L. arcana* and *L. compressa* but not *L. saxatilis*, although rare instances of haplotype sharing between the three *Littorina* species have been found and are likely due to incomplete lineage sorting (Panova *et al.*, in preparation). The constructed priors were normally distributed with mean = 3 Ma and SD = 0.5 Ma (so that 95% of prior distribution lays between 2 and 4 Ma) for the first calibration point and log-normally distributed with mean = 0.5 Ma and SD = 0.5 Ma for the second calibration point (log-normal shape was chosen to include prior values as low as zero and as high as 2 Ma at low probability, thus accounting for uncertainty in divergence time estimates based on allozyme variation, and at the same time to avoid negative values).

We implemented the same model of sequence evolution as in the Bayesian phylogenetic analyses and a relaxed molecular clock with uncorrelated rates among lineages. To choose the coalescence model with the best fit to the data, we tested four possible models: constant population size, exponential growth, population expansion, and Bayesian skyline. We then compared model likelihoods by calculating Bayes Factors (BF, the ratio of the marginal likelihoods of two models). Marginal likelihoods were estimated as harmonic means of the sampled likelihoods in Tracer v. 1.5 [29], [31]. Two independent runs of 50 million generations, with sampling every 10 000 generations and removing 10% of the initial samples as burn-in, were analyzed in LogCombiner v.1.5.2, Tracer v.1.5, and TreeAnnotator v.1.5.2 [29]. Effective sample sizes (ESS) in all runs were above 300.

In addition, we used the molecular clock to calculate the mean age of multiple tip haplotypes, which form star-like phylogenies in the tree. These haplotypes were separated from their central haplotype by one transition in the third codon position; the rate of this class of mutations for the genus *Littorina* in the cytochrome *b* gene has been estimated to be 7.6% per site per Ma [28].

### Genetic diversity and population structure

Haplotype diversity *h* and nucleotide diversity π were estimated using Arlequin 3.01 [32]. We calculated fixation indices for population pairs, based on pairwise differences between haplotypes (*Φ*_ST_), and tested significance of differentiation in Arlequin. Hierarchical analysis of molecular variance (AMOVA) was used to estimate variation among two (“NWA” and “NEA+ISL”) or three regions (“NWA”, “NEA” and “ISL”).

In addition to *a priori* division of populations into regions, we explored spatial relationships among populations using spatial analysis of molecular variance (SAMOVA) in SAMOVA 1.0 [33]. This analysis helps determine whether more variation can be explained by other significant groupings of populations than our *a-priori* divisions. Based on *Φ*-statistics and a simulated annealing algorithm, this method divides all populations into *k* groups, maximizing variation among groups and minimizing variation among populations within groups. We tested divisions from 2 to 15 groups and ran 100 initial conditions to ensure that the algorithm converged. For population divisions found at each *k* level, the significance of the *Φ*-statistic between groups was assessed by non-parametric randomization tests using 10 000 permutations. The grouping of populations explaining the largest proportion of total variation was chosen by examining the pattern of changes in *Φ*-statistics at different *k* levels.

Isolation-by-distance patterns in population structure for both our *a priori* and identified SAMOVA groups were tested by calculating the Mantel *r* correlation between the linearized *Φ*_ST_ values = *Φ*_ST_/(1−*Φ*_ST_) and log-transformed geographic distances between samples (calculated as surface distances from the latitude and the longitude); significance was assessed in Arlequin.

### Demographic reconstructions

In populations that have recently undergone rapid expansion, the distribution of pairwise genetic differences between sequences (mismatch distribution) is expected to be unimodal [34]. We calculated mismatch distributions for population expansion in Arlequin using the groups identified in the SAMOVA analyses. Observed distributions, calculated in Arlequin, were plotted and the shape was inspected by eye (results not shown). The fit of observed mismatch distribution to that expected under the sudden expansion model was further assessed by calculating sums of squared deviations (SSD) between observed and expected values and raggedness index *r* [35], and then comparing them to the values obtained by 1000 random permutations (where significant *P*-values indicate deviation of the data from the expansion model). In addition, Tajima's and Fu's neutrality tests were conducted in Arlequin to provide support for possible population expansion, detected by the mismatch analysis. In the absence of selection, significant negative values of Tajima's *D* and Fu's *F* statistics indicate historical population growth. For the population groups, for which a sudden expansion could not be rejected, time of the start of expansion (*t* in generations) was calculated from the expansion parameter τ using the formula *t* = τ/2*u*, where *u* = 2*μk* (*μ* is a mutation rate per site, *k* is the sequence length). Based on mutation rates of 2 and 4% per site per Ma [25] and 607 bp sequence length, *u* is 24.3–48.6 Ma; *t* in generations was converted to time in years (T) using generation time estimates of 0.5–1 year [36].

Population divergence estimates were performed using the Isolation with Migration-analytical (IMa) program (September 2009 version; [37], [38]). IMa is a coalescent-based method that uses Markov chain Monte Carlo sampling and applies the isolation with migration model to estimate the time of divergence (t), genetic diversities (θ_1_, θ_2_, and ancestral θ_A_), and migration rates (m_1_/μ and m_2_/μ) between two populations assumed to have shared a common ancestor. We performed 2–3 replicate runs of each comparison, which included 30 chains of at least 2 million steps per chain after an initial burn-in period of 100 000 steps; we ended runs when ESS (effective sample size) values were >50 and posterior density parameter curves were stable. Divergence estimates were rescaled to time since separation in years using substitution rates of 2 and 4% per site per Ma as was discussed earlier for *Littorina* [25], [39] (note: we did not apply the 7.6% per site per Ma rate from [28], since it refers only to transitions in the third codon position, while our whole dataset of sequences also contains other, slower mutation types). To estimate the demography of the species across the Atlantic, we performed several pairs of analyses at the regional level (NEA+ISL vs. NWA; NEA vs. NWA; NWA vs. ISL; and NEA vs. ISL). Further, we tested whether migration rates between mainland Europe and Maritime Canada were higher than between mainland Europe and southern US populations (arbitrarily defined as below 43°N), as expected under the hypothesis of natural stepping-stone migration. Within Europe, we tested connections between mainland Europe and the British Isles as well as between Spain (the ice-free area during the LGM) and the rest of mainland Europe, which likely represent post-glacial habitats. Since we found that some populations in NWA and NEA are genetically closely related to ISL populations (see SAMOVA results below), we also tested pairwise differences among these groupings. Finally, we tested the cryptogenic Mediterranean (Venice) population versus its putative source region, Europe.

To determine whether resulting estimates of effective population size, migration rate, and divergence time were significantly different from one another, we used a probability assessment method [40] based on comparisons of randomly drawn values from resulting marginal distributions. The number of times (out of 1000) that the value from the first distribution was greater than from the second approximates the probability that the first parameter was larger than the second. Significant probabilities were defined as ≥0.85 following [40].

## Results

### Sequence variation

**Figure 2:**
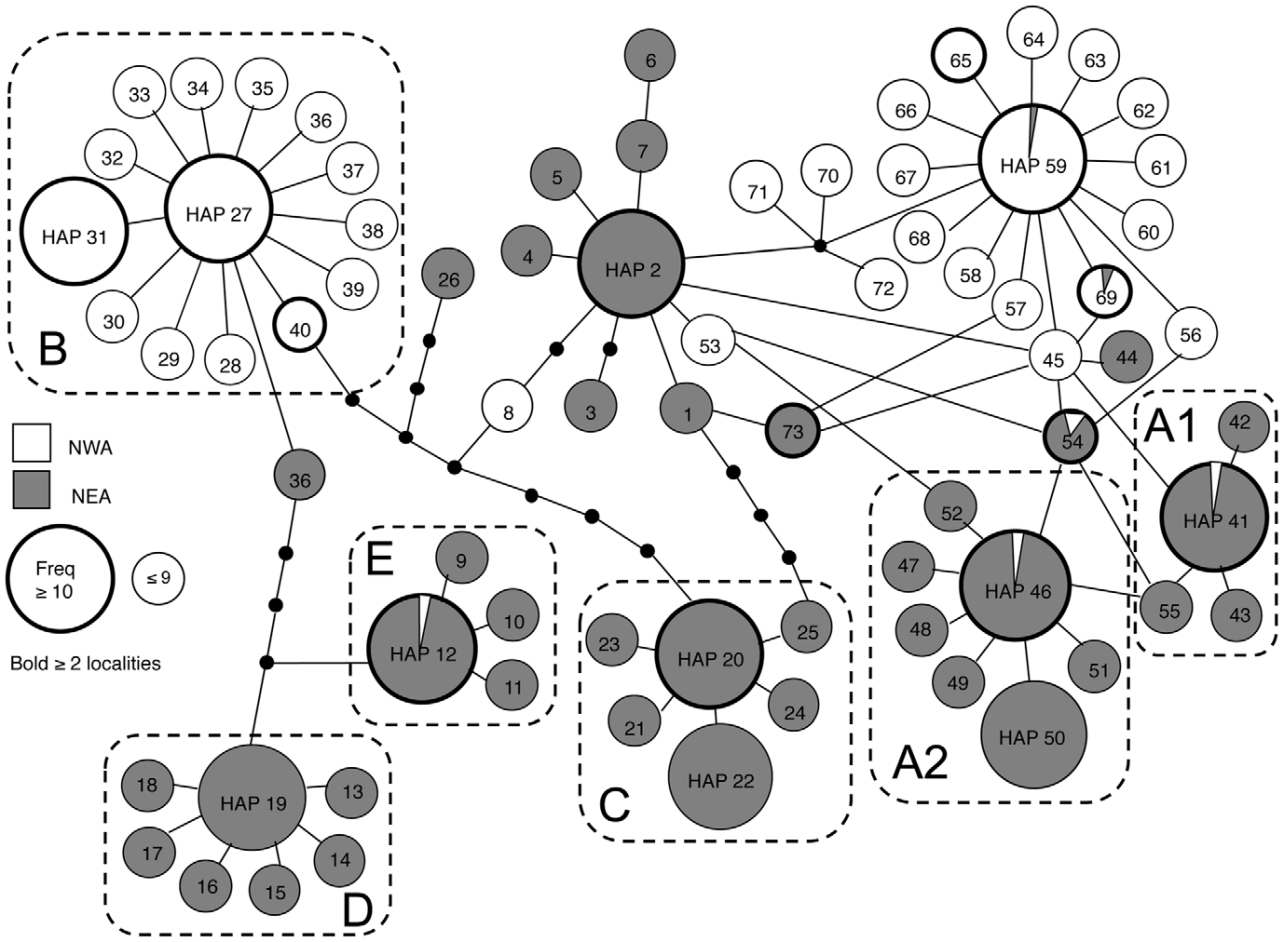
Cytochrome-*b* haplotype network in *L. saxatilis* across the North Atlantic. Those haplotypes restricted to NWA are coloured white; those haplotypes restricted to NEA are coloured gray; and those haplotypes shared across the North Atlantic are displayed as pie-diagrams showing their frequencies in NWA and NEA. Bold circles indicate haplotypes found in more than one locality; the rest are private and found in only one sample. Large bubbles depict haplotypes with frequencies of 10 or more, and small bubbles depict haplotypes with frequencies below 10. Monophyletic clades from a Bayesian phylogenetic analysis are indicated by rounded rectangles; haplotypes outside these rectangles all belong to clade A.

In the analyzed cytochrome *b* fragment we observed 58 variable sites: eight in the first codon position, two in the second and 48 in the third, yielding a high overall mitochondrial DNA diversity (*h* = 0.905; SD = 0.004; π = 0.0099; SD = 0.0052). Most substitutions (52) were silent and only six were non-synonymous. We found a total of 73 haplotypes (Table S2); 32 haplotypes were restricted to NWA, 35 to NEA+ISL and only six were shared between the two major regions (Table S3, Fig. 2). Most of the haplotypes (60 out of 73) were restricted to a single population and only 13 haplotypes were found in more than one location; half of all observed haplotypes (37) were singletons (i.e., found at frequency = 1). However, there were a few haplotypes that were common in several NEA populations (haplotypes 2 and 20), several NWA populations (haplotype 27), or in multiple locations across the Atlantic (haplotypes 12, 41, 46, 59; Table S3). Rarefaction curves for observed and expected diversity in NEA (with or without ISL) and NWA were very similar (Fig. S1) and according to these analyses, additional haplotypes would likely be found with greater sampling effort in both NEA and NWA; however, this “missing” diversity was essentially equivalent in each region. As such, we can be confident that both regions were similarly sampled and that there is no apparent sampling bias in one region versus the other.

### Phylogenetic analyses and geographic distribution of the clades

**Figure 3:**
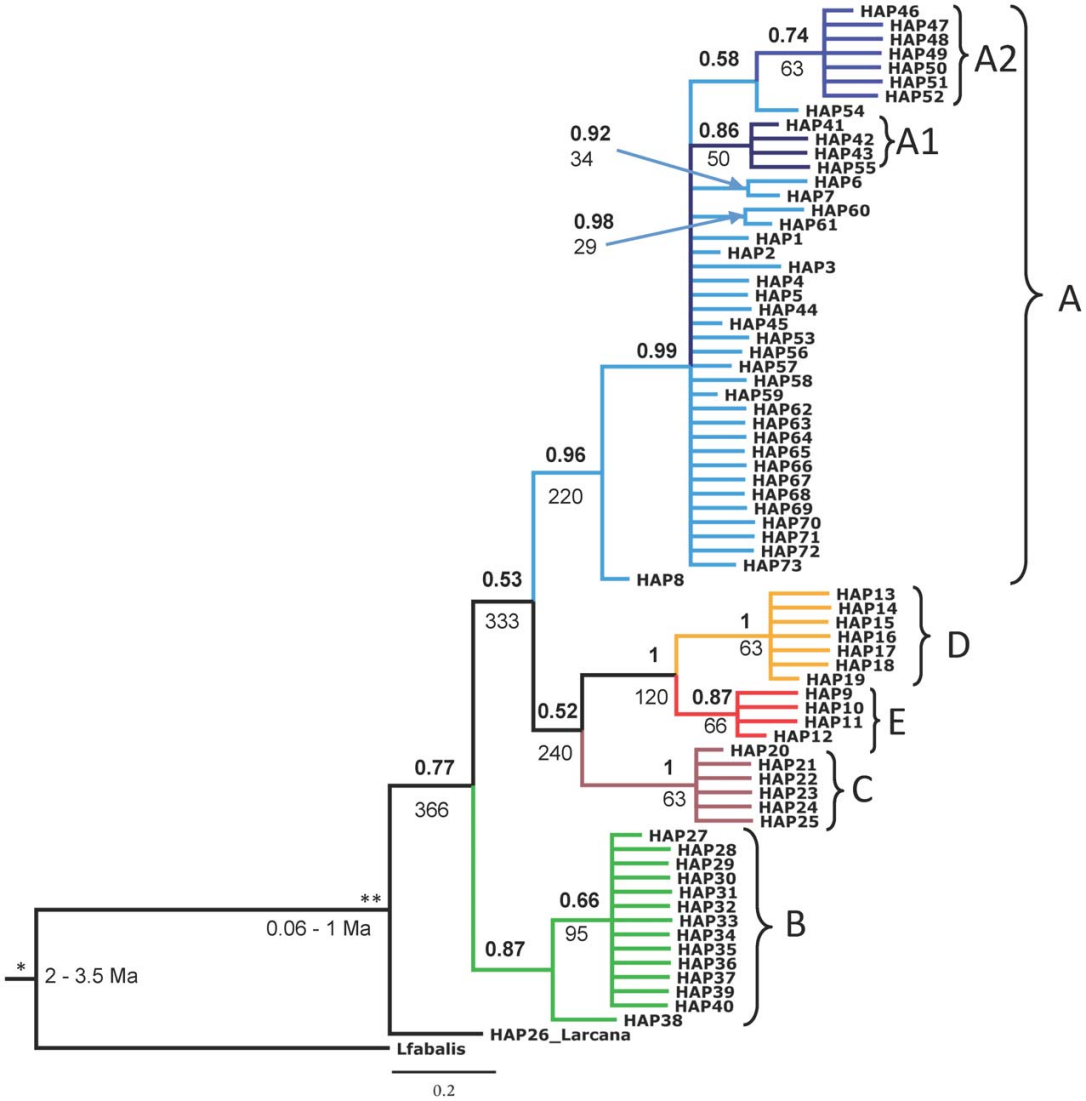
Bayesian phylogenetic tree of *Littorina saxatilis* mitochondrial cytochrome-*b* haplotypes with coalescence times estimated in BEAST. *Littorina fabalis* is included as the outgroup for the tree. Numbers above the nodes refer to posterior probabilities of the clades (probabilities <0.5 are not shown). Clades with posterior probabilities >0.7 (A–E, A1, A2) are indicated by different colours (see Fig. 4 for their frequencies in different populations demonstrated as pie charts). Mean TMRCAs for these clades in ka are given below the nodes, together with the two utilized calibration points: fossils (*) and allozyme divergence (**). Sequence accession numbers are provided in Table S3.

The best substitution model for the analyzed cytochrome *b* fragment, as suggested by Modeltest, was the general time reversible model with gamma-shaped distribution of rates among sites and the proportion of invariable sites (GTR+I+G). This model was subsequently used in Bayesian tree reconstructions. In the Bayesian consensus tree, rooted with an *L. fabalis* sequence, haplotype 26, representing a likely introgression from *L. arcana*, had a basal position in respect to other *L. saxatilis* haplotypes (Fig. 3). The main pattern we observed in *L. saxatilis* cytochrome *b* variation was the existence of several distinct and well-supported clades with some geographical concordance (labelled A-E in Fig. 3). The first, most divergent clade A contained 42 haplotypes, found from northern to southern populations on both sides of the Atlantic. The two monophyletic subclades A1 and A2 are distinguished within this group and found in several sites in Europe (except Spain) and also in Newfoundland and Nova Scotia-1. The rest of clade A consisted of interweaved NWA and NEA haplotypes. The second clade B was found only in NWA. The third clade consisted of three daughter clades: clade C, mainly found in the British Isles; clade D, with highest frequency in Iceland and the Faeroes; and finally, clade E, found only in Spain.

The pattern of the haplotype network is congruent with that also obtained in Bayesian phylogenetic reconstruction (Fig. 2). Each clade has a star-like shape with a single common haplotype connected to a number of rare tip-haplotypes, separated by only one mutational step (Fig. 2). While central haplotypes were present in several geographic locations, the tip haplotypes were generally found in low frequency in single samples (Fig. 2, Table S3).

### Estimated age of mitochondrial lineages

The Bayesian skyline model (BSL), which allows several changes of population size through the species' history, had decisively stronger support than the constant population size model (BF = 613295) and the exponential growth model (BF = 10967), and it had substantially stronger support than the population expansion model (BF = 3.3; interpretation of Bayes Factors according to [41] where values >3 indicate substantial support; values of 30–100 indicate very strong support; and values >100 indicate decisive evidence for the model). Accordingly, we report here the estimation of TMRCA for mitochondrial DNA lineages in *L. saxatilis* obtained with the Bayesian skyline model (Table 1). The mean age of the tree root (TMRCA for *L. saxatilis* and *L. fabalis*) and the mean TMRCA for *L. saxatilis* and *L. arcana* was within the range of initial calibration intervals from fossil and allozyme divergence (Table 1, Fig. 3). Bayesian reconstruction suggests that the evolution of the major mitochondrial lineages in *L. saxatilis* pre-dated the LGM: in that the mean time for major coalescent events was estimated between 366 and 220 kya (Table 1, Fig. 3). Interestingly, the oldest mitochondrial lineage in *L. saxatilis* was the clade B, which was found exclusively in NWA. It is less clear whether the common ancestor of the clades D and E existed before or after the LGM since the 95% HPD interval includes estimates as low as 20 ka. Mean TMRCAs for tip haplotypes in each of the clades (A1, A2, B, C, D and E) ranged from 50 to 95 ka; however, all 95% HPD intervals include possibilities for post-glacial expansion of 13–17 kya (Table 1).

**Table 1:** Divergence time estimates for mitochondrial cytochrome-*b* lineages in *Littorina saxatilis* using the Bayesian Skyline coalescent model in BEAST.

Node	Mean TMRCA (ka)	95% HPD (ka)
((*L. saxatilis*, *L. arcana*), *L. fabalis*))	2500	1400–3500
(*L. saxatilis*, *L. arcana*)	410	150–710
*L. saxatilis*, all clades (B, (A+C+D+E))	366	110–640
Clades A+C+D+E	333	99–618
Clades C+D+E	240	50–470
Clade A	220	40–420
Clades D+E	120	20–220
Clade B	95	17–182
Clade E	66	15–103
Clade C	63	15–188
Clade D	63	14–99
Subclade A2	63	16–97
Subclade A1	50	13–93
Haplotype 6 + Haplotype 7	34	6–76
Haplotype 60 + Haplotype 61	29	1–58

Clades and haplotypes correspond to those in Fig. 3. The tree was calibrated using divergence estimates between *L. saxatilis* and two other species (*L. fabalis* and *L. arcana*). Mean estimates and 95% highest posterior density interval (HPD) for time to most recent common ancestor (TMRCA) of each clade are given in ka (thousands of years).

The youngest splits in the tree (common ancestors for haplotypes 6–7 and 60–61) were dated even closer to the LGM: with a mean of 29–34 ka (1–76 ka) from the coalescent estimation in BEAST; when based on the mutation rate of 7.6% per site per Ma (for transitions in third-codon positions of cytochrome *b* in *Littorina* [28]), the average time for these mutation events is 33 ka.

### Genetic diversity within populations

Most of the populations had high haplotype diversity, ranging from 0.125 to 0.771 (Table S1). Nucleotide diversity was highest in the samples from England, Wales, Ireland-1 and -2 and Iceland in NEA and from several sites in Maine and Massachusetts in NWA. Low nucleotide diversity was found in populations that only had haplotypes from one clade: Spain, Germany, and Mass-3 (see Table S1). Notably, the Venice sample was monomorphic for a haplotype that was common in the British Isles and Sweden. We found no correlation of any diversity measure with latitude within NWA (*r* ^2^ = 0.008, *P* = 0.74 for *h*, *r* ^2^ = 0.037, *P* = 0.47 for π) or within NEA, excluding the Mediterranean monomorphic sample (*r* ^2^ = 0.167, *P* = 0.10 for *h*, *r* ^2^ = 0.017, *P* = 0.62 for π). Private haplotypes (i.e., haplotypes present in only one population) were found in many populations from all clades on the gene tree (Table S3).

### Population structure

There was high genetic differentiation (*Φ*_ST_) between populations of *L. saxatilis*, and 458 of 561 total pair-wise comparisons were statistically significant after sequential Bonferroni correction (Table S4). *A-priori* division of populations into two regions, “NWA” vs. “NEA+ISL,” tested with AMOVA, although significant, explained only 12.6% of the total variance, while variation among populations within regions was 45.2% (Table 2). Separating “ISL” populations into their own group only decreased the *Φ_CT_* value among groups while increasing the variation among populations within groups (Table 2). Consequently, a search for an alternative partition of local populations without *a-priori* assumptions was performed using SAMOVA. Increasing the number of groups from 2 to 7 led to increasing *Φ_CT_* values, and the highest *Φ_CT_* = 0.52 was observed for 7 groupings. The combination with 8 groups produced a slightly lower *Φ_CT_*; and further increases of *k Φ_CT_* values approached a plateau and new groups always contained a single population. The differentiation between groups in the division of seven was highly significant and explained 52% of total variance while variance among populations within the groups was only 6% (Table 2). Thus, we concluded that the division of populations into seven groups best described the high-level population structure in our dataset.

**Table 2:** Analysis of molecular variance (AMOVA) and spatial analysis of molecular variance (SAMOVA) for cytochrome-*b* variation in *Littorina saxatilis*.

Source of variation	df	% Total variance	Fixation indices	*p*-value
**AMOVA, “NWA, NEA+ISL”**				
Among groups	1	12.61	*Φ*_CT_ = 0.126	0.0017
Among populations within groups	32	45.19	*Φ*_SC_ = 0.517	<0.0001
Within populations	744	42.20	*Φ*_ST_ = 0.578	<0.0001
Total	777			
**AMOVA, “NWA, NEA, ISL”**				
Among groups	2	7.76	*Φ*_CT_ = 0.078	0.028
Among populations within groups	31	48.26	*Φ*_SC_ = 0.523	<0.0001
Within populations	744	43.98	*Φ*_ST_ = 0.560	<0.0001
Total	777			
**SAMOVA, 7 groups**				
Among groups	6	52.02	*Φ*_CT_ = 0.520	<0.0001
Among populations within groups	27	6.11	*Φ*_SC_ = 0.127	<0.0001
Within populations	744	41.87	*Φ*_ST_ = 0.581	<0.0001
Total	777			

**Figure 4:**
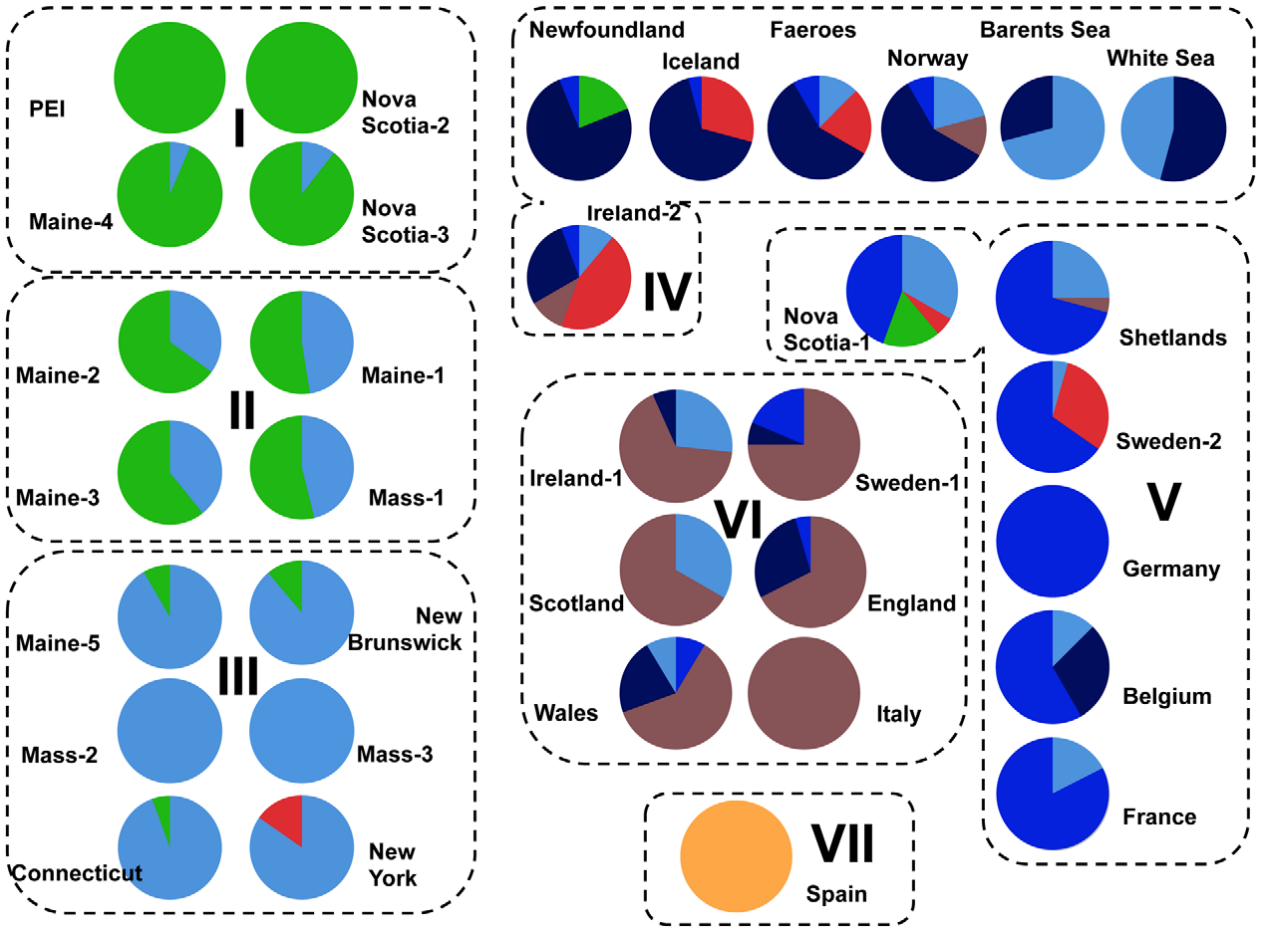
Large scale population structure in *Littorina saxatilis*. SAMOVA partitioning of *Littorina saxatilis* populations into seven groups (I–VII) and frequencies of the cytochrome *b* haplotype clades in the sampled populations (clade colours are as in Fig. 3).

In AMOVA, populations were divided into two groups: NWA (Northwest Atlantic) and NEA (Northeast Atlantic mainland) combined with ISL (North Atlantic Islands) or into three groups: NWA, NEA and ISL. In SAMOVA, results are shown for a partitioning that maximized the percentage of total variance explained by variation among groups (see Fig. 4).

In this partition of seven groupings, NWA populations were divided into three groups (I, II, III) corresponding roughly to their geographical location along the coastline, northeast to southwest. This also coincided with the relative frequencies we observed in mitochondrial clades A and B: the former being more common in the south and decreasing in frequency north with the latter showing the opposite cline (Fig. 4). Interestingly, two of the most northeastern sites in NWA (Newfoundland and Nova Scotia-1) were clustered with some NEA populations. These two populations had haplotypes from lineage B, characteristic for NWA populations (27, 36) but also haplotypes otherwise found in several European populations (41, 46, 54).

The NEA and ISL populations were divided into four groups (IV–VII, Fig. 4). Group IV included Iceland, Faeroes and the northern-most sites in Europe, together with Ireland-2 and Newfoundland. Group V included the Shetlands and more southern mainland European sites (Sweden-2 to France) as well as Nova Scotia-1. Group VI comprised mainly populations from the British Isles but also Sweden-1 and the Venice population. Finally, the Spanish population formed its own group (VII).

We did not observe any isolation-by-distance patterns within NEA, NWA nor SAMOVA groups (Mantel correlation *P*>0.05) except for SAMOVA group IV. Interestingly, this one group showed significant correlation between pairwise genetic differentiation and geographic distances (Mantel *r* = 0.379; *P* = 0.046) and included populations from Newfoundland, Iceland, Faeroes, Ireland-2 and three of the most northern NEA populations (Fig. 4); thus this result may be reflective of stepping-stone dispersal across northern populations in the North Atlantic.

Overall, the SAMOVA partitioning of populations into groups correlated with several geographic boundaries between northern and southern, or island and mainland populations, but also showed some unexpected connections. Importantly, these groupings also coincided with the geographic distribution of the haplotype clades (Fig. 4): clade E and subclade A1 were predominantly found in group IV, subclade A2 had highest frequencies in populations from group V; group VI was dominated by the clade C and finally, the single Galician population forming group VII was monophyletic for the clade D.

### Demographic history

Neutrality tests, SSD-test and raggedness index (Table 3) and unimodal mismatch distributions (results not shown) provided corroborative evidence for population expansion for the Spanish population (i.e. group VII) and group III, which contained six southern NWA populations. Time of expansion was estimated as 6–22 ka for the Spanish population and 9–36 ka for NWA group III. For the other groups, the mismatch distribution was bimodal (results not shown); however, this does not necessarily exclude expansion of these populations, but may reflect the recent colonization by two divergent lineages [42].

**Table 3:** Mismatch analysis and neutrality tests for population groups I-VII of *Littorina saxatilis*, identified by SAMOVA.

	I	II	III	IV	V	VI	VII
Tajima's D	**−1.49** (0.046)	0.79 (0.829)	**−1.55** (0.033)	−0.31 (0.438)	−1.04 (0.144)	0.41 (0.726)	**−1.73** (0.015)
Fu's statistic	−1.54 (0.239)	0.68 (0.657)	**−6.54** (0.009)	−2.27 (0.272)	−4.20 (0.093)	1.93 (0.779)	**−5.27** (<0.001)
SSD	0.020 (0.034)	**0.162** (0.095)	**0.020** (0.254)	**0.052** (0.143)	0.512 (<0.001)	0.458 (<0.001)	**0.005** (0.296)
Raggedness index	0.16 (0.023)	0.23 (0.034)	**0.10** (0.734)	**0.11** (0.091)	**0.09** (1.000)	**0.12** (0.999)	**0.16** (0.356)
τ	NA	NA	0.875	NA	NA	NA	0.529
T (ka)			9–36				6–22

For each statistic, *P*-values obtained by 1000 permutations, are given within parentheses. Values supporting population expansion (i.e. significant negative values of Tajima's D and Fu's statistics and non-significant sum of square deviations between observed and expected mismatch distributions (SSD) and raggedness index *r*) are highlighted in bold. For groups with support for population expansion, time of expansion T (in thousands years, ka) was calculated from expansion parameter τ, mutation rate of 2 and 4% per site per Ma and generation time 0.5–1 years.

IMa results (with 90% highest posterior density (HPD) intervals) for various regional and subregional comparisons are summarized in Table 4 and pairs of parameters found to be significantly different from one another (using the method from [40]) are highlighted in Table S5. Below, we note several of the more important results of these various comparisons. First, regional level comparisons of NEA and NWA suggested that both regions had similar effective population sizes [θ_NEA_ = 41 (HPD 24–60); θ_NWA_ = 45 (HPD 26–66)] and migration rates, estimated as m/μ, between the two regions were low, with no significant difference in directionality (m_NEA_→_NWA_ = 0.22 (HPD 0.01–0.41) versus m_NWA_→_NEA_ = 0.14 (HPD 0.01–0.27)]. Mean divergence estimates (t) (calibrated with mutation rates of 2 and 4% divergence per Ma) ranged from 91–182 (HPD 24–320) kya. Comparisons of NEA+ISL versus NWA regions for all the parameters produced results very similar to those above (Table 4; Table S5).

**Table 4:** IMa estimates of effective population sizes, migration, and divergence time between regional groups of *Littorina saxatilis*.

Pairwise model	IMa parameter results					Divergence time, ka	
	θ_1_	θ_2_	θ_A_	m_1→2_/μ	m_2→1_/μ	2% per Ma	4% per Ma
NEA+ISL vs NWA	50 (32–67)	45 (27–62)	36 (12–60)	0.21 (0.01–0.40)	0.12 (0.01–0.22)	178 (46–334)	89 (23–167)
NEA vs NWA	41 (24–60)	45 (26–66)	38 (12–63)	0.22 (0.01–0.41)	0.14 (0.01–0.27)	182 (48–320)	91 (24–160)
NEA vs ISL	68 (33–158)	6 (3–33)	33 (10–58)	3.12 (0.01–7.69)	3.97 (0.36–7.36)	59 (6–121)	30 (3–61)
NEA(S) vs ISL	29 (20–75)	7 (2–33)	17 (5–45)	3.66 (0.01–7.74)	2.07 (0.01–6.02)	24 (3–91)	12 (1–45)
NWA vs ISL	46 (23–68)	16 (7–25)	20 (5–40)	0.20 (0.10–0.46)	0.32 (0.01–0.56)	68 (22–200)	34 (11–100)
NWA(S) vs ISL	20 (2–28)	14 (5–23)	29 (5–57)	1.79 (0.01–4.76)	3.29 (0.01–6.67)	45 (4–172)	23 (2–86)
NEA(MAIN) vs BI	15 (7–24)	26 (11–40)	24 (10–50)	1.91 (0.17–3.53)	0.74 (0.01–1.64)	51 (14–231)	26 (7–115)
NEA(MAIN) vs SPAIN	18 (9–30)	7 (3–17)	22 (5–60)	0.28 (0.01–0.63)	0.10 (0.01–0.23)	92 (30–498)	46 (15–249)
NEA(MAIN) vs CAN	18 (7–37)	13 (6–33)	26 (1–55)	1.07 (0.23–1.91)	0.16 (0.01–0.37)	76 (14–231)	38 (16–156)
NEA(MAIN) vs USSOUTH	18 (8–40)	25 (11–48)	18 (1–60)	0.16 (0.01–0.36)	0.13 (0.01–0.28)	160 (40–326)	80 (21–163)
NEA+ISL vs VENICE	100 (60–150)	0.2 (0.1–1.6)	34 (16–56)	4.12 (0.01–8.31)	7.15 (0.03–13.62)	5 (0.8–10)	3 (0.4–5)
EUROPE(S) vs VENICE	38 (8–88)	0.1 (0.02–0.3)	25 (15–35)	18 (6–30)	13 (0.02–26)	3 (1–5)	1 (0.6–2)

θ is effective population size (θ = 4Nμ) for first (θ_1_) and second (θ_2_) populations in the model and their ancestral population (θ_A_); migration rates m_1→2_/μ and m_2→1_/μ are from first to second and second to first population in the model, respectively; divergence time is converted to thousands years (ka) using mutation rates of 2 and 4% per Ma. Values in parentheses represent 90% HPD confidence intervals for each parameter (see Table S3 for probability analysis of significant differences between parameters).

Groups of populations are as following (see map in Fig. 1):

NEA – Northeast Atlantic; ISL - Northeast Atlantic islands; NWA – Northwest Atlantic; NEA(S) includes NEA sites grouped in SAMOVA with North Atlantic islands (Ireland-1, Norway, Barents Sea, White Sea, Sweden-2, Germany, Belgium and France); NWA(S) includes NWA populations grouped in SAMOVA with North Atlantic islands (Cape Breton in Nova Scotia and Newfoundland); NEA(MAIN) includes mainland sites in Europe; BI includes all British Isles sites; CAN includes all Maritime Canada sites; USSOUTH includes southern US sites below 43°N; EUROPE(S) includes European sites (British Isles and Sweden) that formed a group with Venice in SAMOVA analyses; SPAIN is the site in Galicia; VENICE is the Venetian lagoon site.

Second, comparisons of the ISL region versus NEA, NWA and subsets of populations grouped with ISL populations in SAMOVA [NEA(S), NWA(S)], demonstrated an effective population size for ISL that was significantly lower than all compared regions. In addition, migration rates between ISL and NEA, NEA(S) and NWA(S) [e.g., m_NEA(S)_→_ISL_ = 3.66 (HPD 0.01–7.74); m_ISL_→_NWA(S)_ = 3.29 (HPD 0.01–6.67)] were an order of magnitude higher than migration rates between NWA and NEA (shown above). However, there was no significant directionality in migration rates between ISL and any of the compared regions. Divergence times between ISL and the compared regions were significantly lower [e.g., t_NEA(S)-ISL_ = 12–24 (HPD 1–91) kya; t_NWA(S)-ISL_ = 23–45 (HPD 2–172) kya] than for NEA-NWA and included the post-glacial time period (Table 4; Table S5).

Third, comparisons between mainland Europe [NEA(MAIN)] and two groups of NWA populations—a) Maritime Canada (CAN) and b) Southern United States (USSOUTH)—revealed significantly higher migration from NEA(MAIN) to CAN than NEA(MAIN) to USSOUTH [m_NEA(MAIN)_→_CAN_ = 1.07 (HPD 0.23–1.91) versus m_NEA(MAIN)_→_USSOUTH_ = 0.16 (HPD 0.01–0.36)]; however, migration rates from both North American regions to Europe were relatively low (m_CAN_→_NEA(MAIN)_ = 0.16 (HPD 0.01–0.37); m_USSOUTH_→_NEA(MAIN)_ = 0.13 (HPD 0.01–0.28)].

Fourth, within NEA, comparisons between NEA(MAIN) and the British Isles (BI) and Spanish (SPAIN) populations revealed an effective population size for NEA(MAIN) that was significantly higher than SPAIN but significantly lower than BI [θ_NEA(MAIN)_ = 15–18 (HPD 7–30); θ_SPAIN_ = 7 (HPD 3–17); θ_BI_ = 26 (HPD 11–40)]. Migration was significantly higher from NEA(MAIN) to BI than from BI to NEA(MAIN) [m_NEA(MAIN)_→_BI_ = 1.91 (HPD 0.17–3.53) versus m_BI_→_NEA(MAIN)_ = 0.74 (HPD 0.01–1.64)], and generally low between NEA(MAIN) and SPAIN [(m_NEA(MAIN)_→_SPAIN_ = 0.28 (HPD 0.01–0.63) versus m_SPAIN_→_NEA(MAIN)_ = 0.10 (HPD 0.01–0.23)]. Estimated divergence time for NEA(MAIN)-BI was also significantly lower than NEA(MAIN)-SPAIN [t_NEA(MAIN)-BI_ = 26–51 (HPD 7–231) kya versus t_NEA(MAIN)-SPAIN_ = 46–92 (HPD 15–498) kya] (Table 4; Table S5).

Finally, comparisons with our Venetian population (VENICE) and two European groupings—a) NEA+ISL and b) European populations grouped with VENICE in SAMOVA [EUROPE(S)]—showed a very low effective population size for the Venice population [θ_VENICE_ = 0.10–0.21 (HPD 0.02–1.57)], which was also significantly lower than both European groupings. We found high migration rates (the rates between EUROPE(S)-VENICE being in fact higher than for all other comparisons) and no significant directionality in migration. Divergence estimates were significantly lower for EUROPE(S)-VENICE than NEA+ISL-VENICE [t_EUROPE(S)-VENICE_ = 1–3 (HPD 0.62–5) ka versus t_NEA(ISL)-VENICE_ = 3–5 (HPD 0.40–10) ka] as well as all other comparisons, notably NEA(MAIN)-BI and NEA(MAIN)-SPAIN (Table 4; Table S5).

## Discussion

Our extensive dataset of 778 mitochondrial sequences from numerous populations on both sides of the Atlantic as well as 3 North Atlantic islands revealed considerable genetic structure across all regions. In addition, genetic diversity was high on both sides of the Atlantic, signifying (along with other analyses) long-term divergence between the two coasts. Notably, the few connections we did observe between the two regions were across North Atlantic islands. Below, we discuss these results in detail, focusing on the two regions and their connections as well as evidence for the existence of glacial refugia in each region.

### High genetic diversity and unique mitochondrial alleles suggest a long continuous history of *L. saxatilis* in the Northwest Atlantic

NWA populations of *L. saxatilis* were comprised of two mitochondrial lineages, A and B, with divergence times (estimated as TMRCA for the clades) of 110–640 ka, of which lineage B appears to be the oldest of all *L. saxatilis* mitochondrial lineages and, in our dataset, is found just in North America. Both the A and B lineages possess multiple alleles, forming star-shaped phylogenies (Fig. 2) which is characteristic of recent population expansions. These expansions likely occurred close to the LGM, as dated by the cytochrome *b* mutation rate (33 ka) and mismatch distribution analyses (9–36 ka, Table 3).

While we observed high genetic diversity and numerous private alleles in most NWA populations in both lineages, little of this diversity (6 of 32 haplotypes) was shared with NEA populations. Moreover, the level of NWA diversity was comparable to NEA (32 versus 35 endemic haplotypes). In addition, coalescent estimates of effective population sizes were not lower for NWA than for NEA (Table 4; Table S5), and mean divergence times (based on 2 and 4% per Ma in IMa analyses) for NWA and NEA populations, 91–182 ka, appear to have pre-dated the LGM (although the lower limits of the 90% HPD were close to the LGM: 24–48 ka). Altogether, these data suggest a deep divergence between NWA and NEA populations of *L. saxatilis* and a long continuous history of the species in North America—thus allowing us to reject the hypothesis for population-level extinction during the LGM in the NWA and adding *L. saxatilis* to the list of species that appear to have survived the LGM in NWA refugia (see [7], [8] for other species).

Of the two mitochondrial lineages present in NWA, one (B) is deeply diverged from all alleles found in NEA while the other (A) is closely related to European alleles, strongly suggesting that the dispersal of these lineages across the Atlantic took place at different time periods. At present, the frequencies of these lineages in NWA populations appear to show a strong latitudinal gradient: the A lineage is more frequent in southern populations while the B lineage is more frequent in northern populations (this is also observed in the NWA population structure found in SAMOVA analyses). This result may potentially reflect survival of the lineages in two different NWA refugia, one northern and one southern, followed by a post-glacial expansion along the North American coast. The oldest clade B may have evolved within NWA or it could have been present in NEA in the past. As suggested for other marine intertidal species [6]–[8], Maritime Canada could be a likely glacial refugium for populations of *L. saxatilis*, especially since the frequency of clade B is highest in northeast NWA populations (Fig. 4).

Clade A, on the other hand, is shared between the eastern and western Atlantic, and this suggests a dispersal event (probably in the Late Pleistocene) for the species across the Atlantic. However, even though western and eastern haplotypes in this clade are phylogenetically closely related, most of the NWA haplotypes are neither shared nor descended from European haplotypes (Fig. 2), as would be expected under a recent re-colonization hypothesis [8] and we cannot definitively establish the clade's region of origin. Moreover, the effective population size for the southern US region, where the A lineage is most common, was higher than in mainland Europe, while migration from Europe was found to be low. It is therefore likely that the dispersal of the A lineage across the Atlantic occurred prior the LGM, perhaps during the Eemian interglacial period (131–112 ka). In fact, our IMa analyses suggest long divergence between southern US populations and mainland Europe, approximately 80–180 (HPD 21–326) ka (Table 4), which would overlap with this interglacial period. These results therefore suggest a second area of refugium for *L. saxatilis* in the NWA, which could have been within the southern-most area of the species' present range where the frequency of lineage A is highest (gradually decreasing northwards), and also where haplotype 8, which is basal for clade A and dated to 40–420 ka, is located (specifically, in the southern-most population in Long Island). Even though habitat south of Long Island lack rocky shores, these areas may still have served as possible refugial locations for intertidal species able to utilize soft sediment habitats [6], [8] - indeed, *L. saxatilis* is also known to live in mud-flat and salt-marsh habitats throughout its range [16].

While the majority of haplotypes in clade A are predominantly found in the US South, it is notable that two subclades (A1 and A2) include haplotypes present in the northern NWA, specifically in Atlantic Canadian populations at Newfoundland and Cape Breton Island (Nova-Scotia-1 on Fig. 1). In fact, most of the NWA haplotypes shared with NEA populations (5 of 6) were observed in these two Canadian populations, and these were also clustered with North Atlantic islands (Fig. 4). This result therefore demonstrates a connection between the NEA and NWA across North Atlantic islands. Furthermore, IMa reconstructions revealed significantly higher migration rates and more recent divergence times between these two Canadian populations and North Atlantic islands than between NEA and NWA populations, as well as between Europe and Maritime Canada (Table 4; Table S5). These data provide congruent, strong evidence for natural stepping-stone migration(s) for *L. saxatilis* across the North Atlantic via Atlantic islands. Likewise, these data corroborate earlier evidence showing connections across northern North Atlantic populations in parasite diversity analyses of *L. saxatilis* [43].

### High mitochondrial variation and complex population structure within the Northeast Atlantic as a signature of survival in multiple refugia

We observed significant genetic differentiation among the seventeen NEA populations, which were divided into four groups based on our SAMOVA analyses (groups IV–VII; Fig. 4). These groups were dominated by different mitochondrial DNA lineages and to some extent also correlated with different geographic regions. In particular, the southernmost sample from Galicia was monophyletic for a distinct clade and also had very large divergence estimates and low migration rates between it and the rest of mainland Europe in IMa analyses (and also significantly lower migration rates/significantly larger divergence time than between mainland Europe and the British Isles; Table 4; Table S5); thus these data demonstrate the clear separation between this population and all other mainland European populations. On the other hand, the three other SAMOVA groupings had less defined geographic boundaries; i.e., northern continental sites in Iceland and the Faeroes, British Isles populations (but also a sample from Sweden), and four southern continental sites (but also Shetland). These groups (and single populations within groups) had more than one mitochondrial lineage and most of the populations had high nucleotide diversity (Table S1). Notably, we did not find higher genetic diversity in southern populations, as would be expected under the hypothesis of expansion from south to north. High genetic diversity in virtually all sampled NEA populations, including Northern Europe, suggests that the post-glacial re-colonization of *L. saxatilis* in NEA occurred rapidly and from multiple refugia [6]. Even if our mismatch distribution analyses showed evidence of recent population expansion only for the Galician population (Table 3), our haplotype network (Fig. 2) supports post-glacial expansion in the form of recent private haplotypes and star-shaped phylogenies, dated close to the LGM in all lineages.

Although the existence of several divergent mitochondrial lineages with distinct geographic distributions in the Northeast Atlantic is often a signature of multiple glacial refugia [6], there might be alternative explanations to this observation. First, deep coalescence between lineages does not provide direct evidence for the existence of several refugia: instead, they may have all evolved within high-structured populations in one large refugium [7], [44]. In this scenario, following ice-retreat, small subsamples of lineages, representing only part of the overall diversity, could have been dispersed to different areas thus creating the observed phylogeographic patterns. Given the snail's lack of planktonic dispersal and its observed strong population structure in NEA, we cannot reject this scenario, although it is difficult from our data to single out a likely region for one large refugium. The only population we sampled that possessed all European lineages was from Limerick, Ireland, which is within a region that has previously been identified in other systems as a likely marine refugium (see below). However, high nucleotide diversity could also be a signature of admixture from several refugia [3], [5], [45]; moreover, the number of private alleles (2) in the Limerick sample was not higher than in many other sampled populations. Therefore, though we cannot rule out a single, large refugium in NEA, multiple refugia would seem the more parsimonious explanation for our results given the high diversity and genetic differentiation we observed in multiple regions across Europe.

Second, coalescent estimates of age for mitochondrial lineages, obtained in BEAST, should be treated with caution, since strong population structure in *L. saxatilis* clearly violates the assumption of a single panmictic population in the implemented coalescent models, and might inflate coalescent times between lineages. Still, the intervals for TMRCA for coalescent events among major lineages do not overlap with the LGM and this divergence happened before the recent, likely post-glacial, diversification of multiple tip-haplotypes observed in each lineage. Thus, the major mitochondrial lineages in *L. saxatilis* most likely pre-date the LGM.

Assuming the hypothesis for multiple refugia is more likely, we discuss here in detail the various European lineages within the species' phylogeny and their connections to specific populations, as these may represent potential areas for these refugial populations. In particular, we observed clade C to predominantly occur in populations from the British Isles (Fig. 4). British Isles, particularly southwest Ireland, have been suggested as refugia for other species, like red and brown algae [46], [47], which is supported by geological evidence that this region was unglaciated during the LGM [48]. High genetic diversity has also been observed in several shallow-water species in the English Channel [46], [47], [49] where Hurd Deep might have been a marine lake during the LGM [46]. Notably, our analyses revealed a higher effective population size in British Isles populations than in the mainland Europe, which would support the hypothesis that the British Isles harboured one or several refugia for the species. Even still, migration was found to be higher from the mainland to the British Isles than vice versa, which does not support the British Isles being a main origin of post-glacial re-colonization for the rest of the European coast. Moreover, clade A (shared with NWA) is relatively rare in the British Isles but common in European mainland populations, where we also found two distinct subclades A1, widespread in northern-most populations of Europe and on Iceland and Faeroes (see below), and A2, predominant in mainland European populations. Given their distinct geographic distributions, it seems unlikely that haplotypes from clades A and C originated from the same refugium; the more likely explanation may be two separate refugia, one being the main source for post-glacial expansion in the British Isles and the other in mainland Europe. Since it is unclear to what extent the North Sea was glaciated, there might have been additional boreal refugia along some of the coasts [6], [50].

The last clade includes two daughter lineages, D and E, and the split between the two cannot be reliably dated before the LGM since 95% HPD for their TMRCA includes estimates as low as 20 ka (Table 1). While clade D was present in several geographically remote samples (Iceland, Faeroes, Ireland, south Sweden and Cape Breton in Nova Scotia), clade E was found to be restricted to Spain. Given such distinct geographic distributions, it is again unlikely that both clades recently shared the same LGM refugium. More likely, the divergence of the two clades (and the isolation of the Spanish population) is older than the LGM, which is supported by divergence time estimates between Spain and other European populations (Table 4). The Iberian peninsula, ice-free during the last glacial period, has been identified as a source for post-glacial re-colonization in many terrestrial species [1], [45], and the coast has likely served as a marine refugium [14], [47]. However, Iberian populations of *L. saxatilis* appear to have played a limited role in the recent post-glacial re-colonization of more northerly areas, and this appears also true in other marine species, where unique mitochondrial lineages are found on the Galician coast; e.g., *Fucus serratus* [47], *Pollicipes pollicipes* [51], *Carcinus maenas* [14] and *Neomysis integer* [49]. Such historical and contemporary isolation of the Galician coast may be the result of oceanic barriers [52], [53].

The sister lineage, clade D, was found predominantly in Iceland and the Faeroes. The existence of divergent Icelandic populations of invertebrates in several other species, for example *Arctica islandica* [13], *Idotea balthica* and *Semibalanus balanoides* [8], as well as a distinct mitochondrial lineage observed in our *L. saxatilis* data from these regions, suggest that Iceland or the Faeroes may have also served as a glacial refugium for *L. saxatilis*. However, observed genetic divergence of Icelandic populations could be also explained by recent non-random colonization of Iceland and the Faeroes from continental populations [15]. The extent of the ice sheets in the North Atlantic remains uncertain; there is geological evidence of solid icecaps covering Iceland and the Faeroes during the LGM [54], but according to some models (see Fig. 1 in [6]), the Faeroes might only have had a small icecap and thus could have harboured refugial populations of intertidal species. The close phylogenetic relationships between the Galician and Iceland/Faeroes lineages in *L. saxatilis* may suggest that both populations are descendants of one interglacial population that became fragmented during the LGM; in fact, phylogenetic connections between Iberian and Northern European populations, dated to the Holsteinian or the Eemian interglacials, have also been observed in the mysid, *Nemysis integer* [49].

### The Venetian population is a recent introduction

The only population that we have clearly identified as a recent and likely anthropogenic introduction is the outlying population in the lagoons of Venice: all individuals had the same haplotype, which was common in several other NEA populations. In addition, estimated effective population size for the Venice population was very low (significantly lower than other European populations) and mean divergence estimates between Venice and NEA collectively, as well as a subset of NEA grouped together with Venice in SAMOVA (see Table 4 and Fig. 4), ranged from 1–5 ka with some estimates as low as 400 years ago. These divergence estimates were significantly lower than estimates for mainland Europe versus British Isles and mainland Europe versus Spain, for example (Table 4; Table S5). However, it should be noted that the model implemented in IMa may not represent the most likely population scenario – i.e., a founder event with a severe bottleneck, perhaps colonization by a single brooding female—as such, divergence time estimates using IMa could very well be inflated. Together with earlier findings of exceptionally low variation in allozyme loci for the Venice population [55], we reject the hypothesis of an ancestral Mediterranean population. A possible introduction vector could have been ship's ballast: the haplotype found in Venetian *L. saxatilis* had its highest frequency outside of Venice in the British Isles, and London and Southampton were main destination ports outside the Mediterranean for merchant galleys of the Venetian Republic in the 15^th^ century [56]. Curiously enough, *L. saxatilis* was first described by Olivi in 1792 based on a specimen from this Venetian lagoon [16]; this record, therefore, provides the minimum age estimate for this population.

### Understanding dispersal mechanisms and phylogeography in *Littorina saxatilis*

The most characteristic signature of glacial refugia is high genetic diversity and the presence of private haplotypes/alleles, in contrast to recently colonized areas that have lower diversity and/or only common haplotypes [42], [57]. Surprisingly, almost all of our sampled populations of *L. saxatilis* had high nucleotide diversity and private haplotypes, making identification of possible refugia challenging. This suggests that after glacial melt, *L. saxatilis* re-colonized habitats very rapidly - in fact, our results show that virtually all populations of the snail underwent rapid increases in population size after the LGM. Thus, despite its lack of pelagic larvae, *L. saxatilis* is actually a very effective coloniser, supporting the hypothesis that establishment of new populations in remote habitats may be facilitated for those species dispersing via egg clutches or brooding females rather than by pelagic larvae [58].

Natural long-distance dispersal in *L. saxatilis* has been hypothesized to occur via rafting or birds [16], [58]; yet direct observations or experimental data are lacking. As such, explicit knowledge of the snail's dispersal mechanisms and frequency of long-distance dispersal events are needed to model the most likely direction of colonization, and to pinpoint the location of post-glacial refugia more precisely. The snail has also been transported long distances anthropogenically (e.g., San Francisco Bay; [59]), and cryptic human-mediated invasions from Europe to North America have been suggested as a possibility for *L. saxatilis* [60]. Though possible, such events do not appear to have had a major effect on the population structure of *L. saxatilis* that we observed (except for the outlying Venetian population, see above), since only older, interior haplotypes were found to be shared among populations. Their derived tip-haplotypes were population-specific, indicating that migration occurred some time in the past and pre-dated the species' population expansion. Even so, we did find one disjunct connection (haplotype 69) between a US population in the lower part of the species' range (Groton, Connecticut) with Limerick, Ireland in Europe, which appeared unusual given that that all the other instances of haplotypes shared between NWA and NEA (n = 5) were found in NWA populations from Maritime Canada. Thus, haplotype 69 could potentially represent an unnatural connection via anthropogenic transport; however, further investigations are required to be certain.

### Conclusions

*Littorina saxatilis* appears to have existed in the Northwest Atlantic for a very long time, and according to our dating analyses, its presence in the Northwest Atlantic could have been for as long a period of time as in the Northeast Atlantic. The snail also appears to have had two major dispersal events in the Northwest Atlantic: 1) early in the species' North Atlantic history and 2) later in its history, possibly during the Eemian interglacial. Glacial refugia for the species along the North American coast were likely located in Maritime Canada and possibly close to the southern edge of the species' present-day distribution. In the Northeast Atlantic, our data suggest the existence of several refugia that were probably located on the Galician coast, Iceland/Faeroes, and possibly southwest Ireland and the English Channel. We also determined that the Venetian population in the Mediterranean Sea is the result of a recent, likely anthropogenic introduction. Post-glacial origin from different refugial populations appears to be a key factor in the large-scale population structure of *L saxatilis*, with little gene flow between the regions at present, except for the noteworthy stepping-stone connection we observed across northern populations in NEA and NWA and North Atlantic islands. Overall, our detailed phylogeography of *L. saxatilis* adds an important piece to the understanding of Pleistocene history in North Atlantic marine biota as well as to resolving possible locations within the North Atlantic for glacial refugia during the LGM. Finally, our investigation provides essential information for studies of adaptive variation and the origin of ecotype populations of *L. saxatilis*.

## Supporting Information

Figure S1 **Rarefaction analyses of mitochondrial haplotype diversity with sampling effort in** ***Littorina saxatilis*** **.**
(DOC)

Table S1 **Sampling localities, coordinates, sample size and measures of genetic diversity within sampled populations of** ***Littorina saxatilis*** **.**
(DOC)

Table S2 **Summary of polymorphic sites and unique haplotypes in the cytochrome** ***b*** **fragment in** ***Littorina saxatilis*** **.**
(DOC)

Table S3 **Cytochrome-** ***b*** **haplotype accession numbers and their occurrences in sampled populations of** ***Littorina saxatilis*** **.**
(DOC)

Table S4 **Pairwise** ***Φ*** **_ST_ and tests for significance of differentiation between populations of** ***Littorina saxatilis*** **.**
(XLS)

Table S5 **Probability calculations of pairwise comparisons for IMa parameter estimates.**
(XLS)
