# Supplementary material for: Glacial History of the North Atlantic Marine Snail, Littorina saxatilis, Inferred from Distribution of Mitochondrial DNA Lineages
Source: PLoS One. 2011 Mar 11;6(3):e17511. doi: 10.1371/journal.pone.0017511 (PMC3055875; doi:10.1371/journal.pone.0017511)
Supplement: Figure S1 — Rarefaction analyses of mitochondrial haplotype diversity with sampling effort in Littorina saxatilis . (DOC) [file pone.0017511.s001.doc]

**Figure S1**. Rarefaction analysis of mitochondrial haplotype diversity with sampling effort in *Littorina saxatilis*.


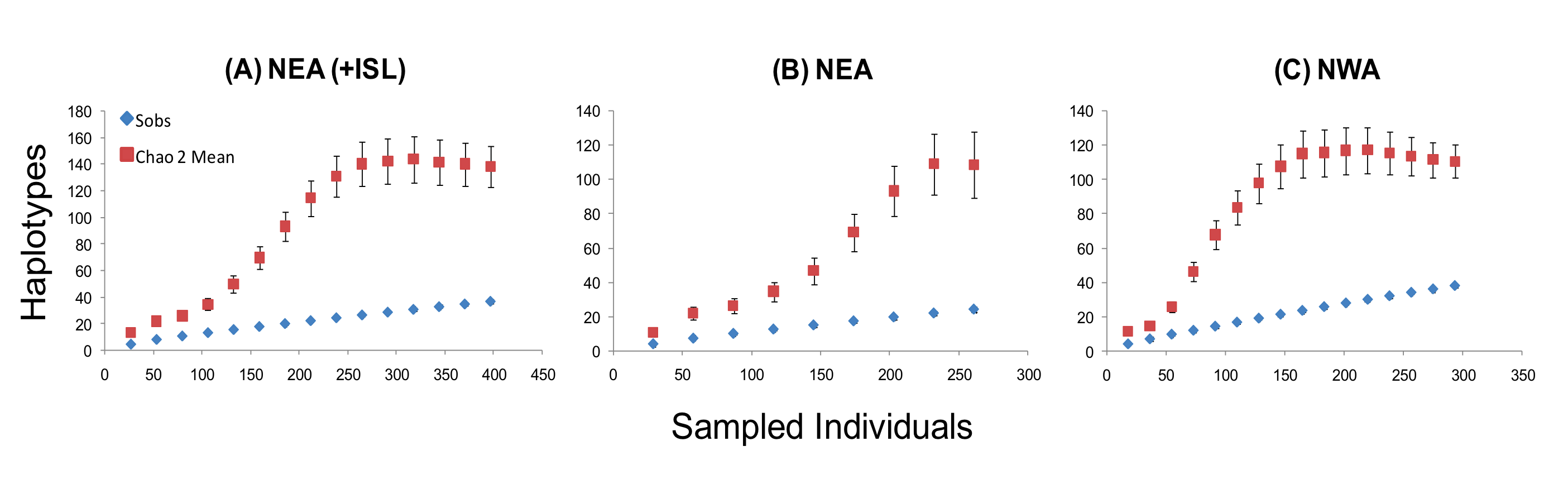


Using EstimateS |24], we constructed rarefaction curves of observed haplotype diversity and estimated diversity (using the Chao2 estimator) to determine effects of sampling on diversity and also to provide estimates of the “missing” haplotypes in our sampling—resulting in expected totals of haplotype diversity at an asymptote. Here, we show three different regional analyses: (A) the Northeast Atlantic combined with North Atlantic islands (NEA + ISL); (B) the Northeast Atlantic (NEA); and (C) the Northwest Atlantic (NWA). Comparing observed and expected curves suggests that more haplotypes are expected in all three regions; however, the “missing” diversity was essentially equal in each region. (e.g., the total expected diversity based on Chao2 was ~140 haplotypes for NEA+ISL and ~120 haplotypes for NEA and NWA). This supports other analyses that show the two regions to have similar levels of genetic diversity and deep divergence between the two Atlantic regions.
