# Supplementary material for: Glacial History of the North Atlantic Marine Snail, Littorina saxatilis, Inferred from Distribution of Mitochondrial DNA Lineages
Source: PLoS One. 2011 Mar 11;6(3):e17511. doi: 10.1371/journal.pone.0017511 (PMC3055875; doi:10.1371/journal.pone.0017511)
Supplement: Table S1 — Sampling localities, coordinates, sample size and measures of genetic diversity within sampled populations of Littorina saxatilis . (DOC) [file pone.0017511.s002.doc]

**Table S1**. Sampling localities, coordinates, sample size and measures of genetic diversity within sampled populations of *Littorina saxatilis*.

| **Name on map** | **Locality** | **Coordinates** | ***N*** | ***a*** | ***b*** | ***h*** | **π** |
| --- | --- | --- | --- | --- | --- | --- | --- |
|  | **NEA** |  |  |  |  | 0.547±0.047 | 0.0048±0.0007 |
| Barents Sea | Chevry, Barents Sea, Russia | 69.2000N; 35.3833E | 24 | 3 | 3 | 0.692 | 0.0022 |
| White Sea | Chupa Bay, White Sea, Russia | 66.3333N; 33.6667E | 24 | 3 | 3 | 0.562 | 0.0018 |
| Norway | Telegraph Bay, Tromsø, Norway | 65.6828N; 18.9428E | 24 | 7 | 14 | 0.761 | 0.0053 |
| Sweden-1 | Ramsholmen, Koster archipelago, Sweden | 58.8500N; 11.0500E | 48 | 3 | 11 | 0.407 | 0.0063 |
| Sweden-2 | Hovs Hallar, Sweden | 56.4722N; 12.7056E | 23 | 4 | 10 | 0.605 | 0.0069 |
| Scotland | Oban, Scotland | 56.4167N; 5.4667W | 9 | 2 | 8 | 0.500 | 0.0066 |
| Wales | Black Rock, SW-Wales, UK | 55.8833N; 4.1667W | 23 | 6 | 13 | 0.731 | 0.0080 |
| Germany | Sylt, Germany | 54.9000N; 8.2833E | 24 | 3 | 2 | 0.304 | 0.0005 |
| Ireland-1 | Sligo, Ireland | 54.4500N; 8.8000W | 15 | 4 | 10 | 0.543 | 0.0070 |
| England | Thornwick Bay, Yorkshire, UK | 54.1333N; 0.1167W | 47 | 6 | 17 | 0.729 | 0.0080 |
| Ireland-2 | Limerick, Ireland | 52.6639N; 8.6278W | 18 | 7 | 15 | 0.771 | 0.0093 |
| Belgium | Zeebrugge Harbour, Belgium | 51.5500N; 3.3333E | 24 | 4 | 5 | 0.630 | 0.0029 |
| France | Carnac, Brittany, France | 47.5833N; 3.1083E | 23 | 3 | 5 | 0.316 | 0.0020 |
| Italy | Chioggia, Venetian lagoon, Italy | 45.2333N; 12.2967E | 40 | 1 | 0 | 0 | 0 |
| Spain | Silleiro, Galicia, Spain | 42.1167N; 8.9000W | 47 | 7 | 6 | 0.407 | 0.0007 |
|  | **ISL** |  |  |  |  |  |  |
| Iceland | Nord∂alur, Iceland | 65.6167N; 23.3833W | 24 | 4 | 11 | 0.533 | 0.0071 |
| Faeroes | Kirkjubøur, Streymoy, Faeroe Islands | 61.9561N; 6.7936E | 24 | 6 | 12 | 0.649 | 0.0067 |
| Shetlands | Hamnavoe, Shetland Islands | 60.1053N; 1.3214W | 24 | 7 | 13 | 0.714 | 0.0045 |
|  | **NWA** |  |  |  |  | 0.484±0.058 | 0.0041±0.0007 |
| Newfoundland | Newfoundland (Bay Bulls), Canada | 47.1837N; 52.4453W | 16 | 4 | 11 | 0.442 | 0.0055 |
| Nova Scotia-1 | Cape Breton, Nova Scotia, Canada | 46.1354N; 60.2917W | 18 | 6 | 12 | 0.758 | 0.0067 |
| PEI | Charlottetown, Prince Edward Island, Canada | 46.1347N; 63.0736W | 17 | 4 | 3 | 0.566 | 0.0011 |
| New Brunswick | Cape Enrage, New Brunswick, Canada | 45.3551N; 64.4653W | 18 | 4 | 11 | 0.399 | 0.0035 |
| Nova Scotia-2 | Halifax, Nova Scotia, Canada | 44.3728N; 63.3349W | 17 | 1 | 0 | 0 | 0 |
| Maine-1 | Jonesport, Maine, USA | 44.3140N; 67.3654W | 21 | 5 | 10 | 0.729 | 0.0079 |
| Maine-2 | Camden, Maine, USA | 44.1348N; 69.0228W | 20 | 4 | 11 | 0.647 | 0.0076 |
| Nova Scotia-3 | Yarmouth, Nova Scotia, Canada | 43.4956N; 66.0722W | 19 | 5 | 10 | 0.386 | 0.0031 |
| Maine-3 | Boothbay, Maine, USA | 43.4738N; 69.5633W | 28 | 5 | 12 | 0.638 | 0.0078 |
| Maine-4 | Portland, Maine, USA | 43.3626N; 70.1258W | 16 | 2 | 9 | 0.125 | 0.0019 |
| Maine-5 | Appledore Island, Maine, USA | 42.5925N; 70.3700W | 24 | 6 | 13 | 0.594 | 0.0033 |
| Mass-1 | Glouchester, Massachusetts, USA | 42.3614N; 70.3920W | 26 | 6 | 13 | 0.689 | 0.0083 |
| Mass-2 | Plymouth, Massachusetts, USA | 41.5948N; 70.3700W | 7 | 3 | 3 | 0.524 | 0.0014 |
| Mass-3 | Martha's Vineyard, Massachusetts, USA | 41.2501N; 70.3301W | 14 | 2 | 1 | 0.143 | 0.0002 |
| Connecticut | Groton, Connecticut, USA | 41.1913N; 72.0000W | 18 | 6 | 12 | 0.719 | 0.0031 |
| New York | Montauk, New York, USA | 41.0413N; 71.5124W | 14 | 3 | 9 | 0.385 | 0.0046 |

*N*- sample size, *h* – haplotype diversity, π - nucleotide diversity, *a* – number of haplotypes, *b* – number of polymorphic sites in the samples.

Mean and S.E. for diversity measures are also provided for NEA+ISL and NWA regions.
