# Supplementary material for: Glacial History of the North Atlantic Marine Snail, Littorina saxatilis, Inferred from Distribution of Mitochondrial DNA Lineages
Source: PLoS One. 2011 Mar 11;6(3):e17511. doi: 10.1371/journal.pone.0017511 (PMC3055875; doi:10.1371/journal.pone.0017511)
Supplement: Table S2 — Summary of polymorphic sites and unique haplotypes in the cytochrome b fragment in Littorina saxatilis . (DOC) [file pone.0017511.s003.doc]

Table S2. Summary of polymorphic sites and unique haplotypes in the cytochrome *b* fragment in *Littorina saxatilis*. (obt – outgroup *L. obtusata*)

1111111111111122222222223333333333444444444444444555555555555556

14455677990012222236888922566677990255677789001112335666789001122333577780

position: 655647659010800236988039525614569479447325770584780250235492140626247536953

HAP1 TCCCCTTTGCAGCGTGCTATATCTTCATGTCAATCAAGTAGATGATATTGTCTTCGTCTAACAATCTACTTCTCC

HAP2 ......C....................................................................

HAP3 .....CC...............................................................C....

HAP4 ......C...............................................T....................

HAP5 ......C.....................................G..............................

HAP6 ......C....A..........T....................................................

HAP7 ......C...............T....................................................

HAP8 ......C........................................C....C......................

HAP9 ....T.C...G..........................A..T......CC...C....T.................

HAP10 ....T.C......A.......................A..T......CC...C....T.................

HAP11 ....T.C....A.........................A..T......CC...C....T.................

HAP12 ....T.C..............................A..T......CC...C....T.................

HAP13 ....T.C......................C.......A..T...........CC...T.................

HAP14 ..T.T.C..............................A..T...........CC...T.................

HAP15 ....T.C..............................AC.T...........CC...T.................

HAP16 ....T.C..............................A..T...........CC..CT.................

HAP17 ....T.C..............................A..T..........ACC...T.................

HAP18 ....T.C..............................A..T........C..CC...T.................

HAP19 ....T.C..............................A..T...........CC...T.................

HAP20 ....T..........................................C....C....T....G..T.........

HAP21 ....T..........................................C....C....T....G..T...C.....

HAP22 ....T......................................A...C....C....T....G..T.........

HAP23 ....T..........................................C....C.T..T....G..T.........

HAP24 ....T..........................................C....C....T.G..G..T.........

HAP25 ....T..........................................C.........T....G..T.........

HAP26 ......C.................C....................C.C....C....T...T.........T...

HAP27 ....T.C...............T.C...............A......C....C....T.................

HAP28 ....T.C..........C....T.C...............A......C....C....T.................

HAP29 ....T.C..T............T.C...............A......C....C....T.................

HAP30 ....T.C...............T.C.....T.........A......C....C....T.................

HAP31 ....T.C...............T.CT..............A......C....C....T.................

HAP32 ....T.C...............T.C...A...........A......C....C....T.................

HAP33 ....T.C...............T.C...............A..A...C....C....T.................

HAP34 ....T.C...............T.C...........G...A......C....C....T.................

HAP35 ....T.C...............T.C...............A......C....C....T...........C.....

HAP36 ....T.C...............T.C............A..A......C....C....T.................

HAP37 ....T.C...............T.C.......C.......A......C....C....T.................

HAP38 ....T.C...............T.C......C...............C....C....T.................

HAP39 ....T.C...............T.C...............A......C....C....T..G..............

HAP40 ......C...............T.C...............A......C....C....T.................

HAP41 ......C..........................C..........................G..............

HAP42 ......C.................C........C..........................G..............

HAP43 ......C..........................C..........................G.....C........

HAP44 ......C................C....................................G..............

HAP45 ......C.....................................................G..............

HAP46 ......C.................................A...................G......G.......

HAP47 ......C........A........................A...................G......G.......

HAP48 ...T..C.................................A...................G......G.......

HAP49 ......C..............C..................A...................G......G.......

HAP50 ......C.................................A...................G......G....C..

HAP51 ......C..............................A..A...................G......G.......

HAP52 ......C.................................A..........................G.......

HAP53 ......C.................................A..................................

HAP54 ......C.................................A...................G..............

HAP55 ......C..........................C......A...................G..............

HAP56 ......C.................................A..................GG..............

HAP57 ...........................................................GG..............

HAP58 ......C..............................A.....................GG..............

HAP59 ......C....................................................GG..............

HAP60 .T....C............................C.......................GG..............

HAP61 ......C............................C.......................GG..............

HAP62 ......C....................................................GG..........T...

HAP63 ......C....................................................GG.............T

HAP64 ......C.A..................................................GG..............

HAP65 ......C....................................................GG....T.........

HAP66 ......C....A...............................................GG..............

HAP67 C.....C....................................................GG..............

HAP68 ......C....................................................GG...C..........

HAP69 ......C....................................................CG..............

HAP70 ......C...........................T........................G...............

HAP71 ......C....................................................G.............T.

HAP72 ......C...................................C................G...............

HAP73 ............................................................G..............

obt ..A...CC....T.C.T.CCG...C.GC.........A.CAG.A..GC..C.C..A.TCG.T.G.T..T......
