# Supplementary material for: Glacial History of the North Atlantic Marine Snail, Littorina saxatilis, Inferred from Distribution of Mitochondrial DNA Lineages
Source: PLoS One. 2011 Mar 11;6(3):e17511. doi: 10.1371/journal.pone.0017511 (PMC3055875; doi:10.1371/journal.pone.0017511)
Supplement: Table S3 — Cytochrome- b haplotype accession numbers and their occurrences in sampled populations of Littorina saxatilis . (DOC) [file pone.0017511.s004.doc]

**Table S3. Cytochrome-*b* haplotype accession numbers and their occurrences in sampled populations of *Littorina saxatilis*.**

| **Haplotype** | **Clade** | **Accession number** | **NEA+ISL populations** | **NWA populations** | **Total** |
| --- | --- | --- | --- | --- | --- |
| Hap1 | A | JF340294 | Barents Sea (8) |  | 8 |
| **Hap2** | A | JF340295 | Barents Sea (9), White Sea (12), Norway (4), France (2), Wales (2), Ireland-1 (1), Shetlands (1), Faeroes (3) |  | 34 |
| Hap3 | A | JF340296 | France (2) |  | 2 |
| Hap4 | A | JF340297 | Norway (1) |  | 1 |
| Hap5 | A | JF340298 | White Sea (1) |  | 1 |
| Hap6 | A | JF340299 | Shetlands (1) |  | 1 |
| Hap7 | A | JF340300 | Shetlands (1) |  | 1 |
| Hap8 | A | JF340301 |  | New York (1) | 1 |
| Hap9 | E | JF340302 | Faeroes (2) |  | 2 |
| Hap10 | E | JF340303 | Iceland (3) |  | 3 |
| Hap11 | E | JF340304 | Faeroes (2) |  | 2 |
| **Hap12*** | E | JF340305 | Sweden-2 (7), Ireland-2 (8), Faeroes (1), Iceland (4) | New Brunswick (1),  New York (2) | 23 |
| Hap13 | D | JF340306 | Spain (2) |  | 2 |
| Hap14 | D | JF340307 | Spain (1) |  | 1 |
| Hap15 | D | JF340308 | Spain (1) |  | 1 |
| Hap16 | D | JF340309 | Spain (1) |  | 1 |
| Hap17 | D | JF340310 | Spain (5) |  | 5 |
| Hap18 | D | JF340311 | Spain (1) |  | 1 |
| Hap19 | D | JF340312 | Spain (36) |  | 36 |
| **Hap20** | C | JF340313 | Norway (3), Sweden-1 (36), Italy (40), Wales (11), England (13), Ireland-1 (10), Ireland-2 (2), Shetlands (1) |  | 116 |
| Hap21 | C | JF340314 | Wales (1) |  | 1 |
| Hap22 | C | JF340315 | England (17) |  | 17 |
| Hap23 | C | JF340316 | England (1) |  | 1 |
| Hap24 | C | JF340317 | England (2) |  | 2 |
| Hap25 | C | JF340318 | Scotland (6) |  | 6 |
| Hap26 | ¶ | JF340319 | England (1) |  | 1 |
| **Hap27** | B | JF340320 |  | Newfoundland (2), Nova Scotia-1 (3), Nova Scotia-3 (15), PEI (11), New Brunswick (2), Maine-1 (6), Maine-2 (10), Maine-3 (14), Maine-4 (15), Maine-5 (2), Mass-1 (11), Connecticut (1) | 92 |
| Hap28 | B | JF340321 |  | Maine-3 (1) | 1 |
| Hap29 | B | JF340322 |  | Maine-2 (2) | 2 |
| Hap30 | B | JF340323 |  | Maine-1 (1) | 1 |
| **Hap31** | B | JF340324 |  | Nova Scotia-2 (17), PEI (3) | 20 |
| Hap32 | B | JF340325 |  | PEI (1) | 1 |
| Hap33 | B | JF340326 |  | PEI (2) | 2 |
| Hap34 | B | JF340327 |  | Mass-1 (2) | 2 |
| Hap35 | B | JF340328 |  | Maine-2 (1) | 1 |
| Hap36 | B | JF340329 |  | Newfoundland (1) | 1 |
| Hap37 | B | JF340330 |  | Nova Scotia-3 (1) | 1 |
| Hap38 | B | JF340331 |  | Maine-3 (2) | 2 |
| Hap39 | B | JF340332 |  | Maine-1 (4) | 4 |
| **Hap40** | B | JF340333 |  | Nova Scotia-3 (1), Maine-1 (1) | 2 |
| **Hap41*** | A1 | JF340334 | Barents Sea (7), White Sea (11), Norway (11), Sweden-1 (3), Belgium (7), Wales (5), England (13), Ireland-1 (1), Ireland-2 (4), Faeroes (14), Shetlands (16) | Newfoundland (12) | 104 |
| Hap42 | A1 | JF340335 | Norway (2) |  | 2 |
| Hap43 | A1 | JF340336 | Norway (1) |  | 1 |
| Hap44 | A | JF340337 | Ireland-2 (1) |  | 1 |
| Hap45 | A | JF340338 |  | Mass-3 (1) | 1 |
| **Hap46*** | A2 | JF340339 | Norway (2), Sweden-1 (9), Sweden-2 (13), Germany (20), Belgium (13), France (19),  Wales (2), England (2), Ireland-2 (1), Shetlands (5), Iceland (1) | Newfoundland (1),  Nova Scotia-1 (8) | 96 |
| Hap47 | A2 | JF340340 | Belgium (1) |  | 1 |
| Hap48 | A2 | JF340341 | Germany (2) |  | 2 |
| Hap49 | A2 | JF340342 | Sweden-2 (2) |  | 2 |
| Hap50 | A2 | JF340343 | Shetlands (12) |  | 12 |
| Hap51 | A2 | JF340344 | Faeroes (2) |  | 2 |
| Hap52 | A2 | JF340345 | Germany (2) |  | 2 |
| Hap53 | A | JF340346 |  | Nova Scotia-1 (1) | 1 |
| **Hap54*** | A | JF340347 | Sweden-2 (1), Scotland (3) | Nova Scotia-1 (1) | 5 |
| Hap55 | A1 | JF340348 | Ireland-2 (1) |  | 1 |
| Hap56 | A | JF340349 |  | Nova Scotia-3 (1) | 1 |
| Hap57 | A | JF340350 |  | Maine-4 (1) | 1 |
| Hap58 | A | JF340351 |  | Maine-4 (1) | 1 |
| **Hap59*** | A | JF340352 | Ireland-1 (3) | Nova Scotia-1 (4), Nova Scotia-3 (1), New Brunswick (14), Maine-1 (9), Maine-2 (7), Maine-3 (10), Maine-4 (1), Maine-5 (15), Mass-1 (10), Mass-2 (5), Mass-3 (13), Connecticut (6), New York (11) | 109 |
| Hap60 | A | JF340353 |  | Maine-5 (1) | 1 |
| Hap61 | A | JF340354 |  | Maine-5 (4) | 4 |
| Hap62 | A | JF340355 |  | New Brunswick (1) | 1 |
| Hap63 | A | JF340356 |  | Connecticut (1) | 1 |
| Hap64 | A | JF340357 |  | New Brunswick (1) | 1 |
| **Hap65** | A | JF340358 |  | Mass-2 (1), Connecticut (1) | 2 |
| Hap66 | A | JF340359 |  | Mass-1 (1) | 1 |
| Hap67 | A | JF340360 |  | Mass-1 (1) | 1 |
| Hap68 | A | JF340361 |  | Maine-3 (1) | 1 |
| **Hap69*** | A | JF340362 | Ireland-2 (1) | Connecticut (8) | 9 |
| Hap70 | A | JF340363 |  | Connecticut (1) | 1 |
| Hap71 | A | JF340364 |  | Maine-1 (1) | 1 |
| Hap72 | A | JF340365 |  | Mass-2 (1) | 1 |
| **Hap73** | A | JF340366 | Belgium (3), Shetlands (3) |  | 6 |

Clade names refer to the clades with high support in Bayesian tree (¶ - haplotype 26 is a likely introduction from *L. arcana*). Names of populations where haplotypes were found are provided together with sampling occurrences in parentheses; for geographic coordinates and location of populations see Fig. 1 and Table 1. The last column is the total occurrence in 778 analyzed individuals. Haplotypes found in more than one populations are in bold and haplotypes shared across the Atlantics are denoted with *.
